# Biological and Chemical Co-surfactant for Fabrication of Antibacterial Silver Nanoparticles and Potential Application in Agriculture

**DOI:** 10.21315/tlsr2025.36.3.6

**Published:** 2025-10-31

**Authors:** Nguyen Nhat Nam

**Affiliations:** Applied Biology Center, School of Agriculture and Aquaculture, Tra Vinh University, No. 126 Nguyen Thien Thanh Street, Vinh Long Province 98000, Vietnam

**Keywords:** Silver Nanoparticles, Antibacterial, Co-surfactant, Biological Surfactant

## Abstract

Silver nanoparticles (AgNPs) have been widely applied as antimicrobial materials. In this work, a new fabrication method of AgNPs has been proposed through a combination of tea seed saponin extraction as a non-ionic biological surfactant and cetyltrimethylammonium chloride (CTAC) as a co-surfactant. The morphology and optical properties of as-prepared AgNPs were analysed by SEM and UV-vis absorbance measurement, respectively. The results indicate that AgNPs obtained high homogeneous particle sizes with a mean diameter of 44.5 ± 3.8 nm. The optical property of AgNPs was exhibited through a UV-vis absorbance spectrum of ~420 nm. In addition, the antibacterial behaviour of *E. coli* (ATCC 25922) was increased according to the AgNPs concentration. The diameter of inhibition zones was 12 mm, 14 mm and 16 mm under AgNPs concentrations of 0.8 ppm, 8 ppm and 80 ppm, respectively. Our initial trial treatment of AgNPs in young broccoli (*Brassica oleracea*) exhibited promising potential for plant protection in agricultural applications.

**HIGHLIGHTS**

- A combination of biological and chemical co-surfactants for the synthesis of silver nanoparticles.
- As-prepared AgNPs showed antibacterial activity against *E. coli*, with inhibition increasing by concentration.
- As-prepared AgNPs treatment improved broccoli health without toxicity, suggesting potential for eco-friendly crop protection.

## INTRODUCTION

Silver nanoparticles (AgNPs) have emerged as antibacterial materials that are less toxic to humans and widely applied in various fields such as food security, biosensors, diagnostics and therapy quality, and crop protection (Husain *et al*. 2023; Rasheed *et al*. 2023). In agriculture, several approach-based AgNPs have been designed and developed for the diagnosis and treatment of crop diseases (Khan *et al*. 2023). It has been reported that AgNPs can positively enhance the growth and development of plants, referring to physiological, biochemical and molecular pathways (Khan *et al*. 2023). For example, Krishnaraj *et al*. (2010) have provided an initial investigation on the effects of AgNPs on the biosynthesis of important metabolites such as carbohydrates and proteins (Krishnaraj *et al*. 2010). AgNPs can take part in the reduction of catalase and peroxidase activities in germination, promoting germination and seedling growth, and inducing plant growth (Hemalatha *et al*. 2024). AgNPs also have been proposed for use as nano-fungicides against early blight in tomato plants. The treated AgNPs tomato crops obtained rapid enhancement in plant height (30%), number of leaves, weight (45%) and dry weight (40%) compared to untreated plants (Ansari *et al*. 2023). The emerging potential application of AgNPs requires updating research on advanced methods for synthesising AgNPs with low cost, eco-friendliness and high efficiency. AgNPs can be fabricated by various methods including “top-down” and “bottom-up”. The green-synthesis or bio-synthesis methods that utilise biological materials such as plant extraction or compounds and microorganisms have been increasing interest due to their match sustainable development and low impact on the environment (Ivanov *et al*. 2023). Phytochemicals and secondary metabolism in plant extraction such as phenolic acid and flavonoids have the ability to convert Ag^+^ to Ag^0^ in order to form AgNPs (Liaqat *et al*. 2022).

Tea seed powder of *Camellia oleifera* has been considered a byproduct remaining residue after extraction of oil and contains a certain saponin substance. The conventional usage of tea tree saponin powder is a natural source of organic fertiliser without taking advantage of saponin contained as abundant low-cost herbal materials. Saponin in *Camellia oleifera* has been investigated to work as a biological non-ionic surfactant and has the capacity of foaming, emulsifying, dispersing, wetting, anti-cancer, anti-inflammatory and antibacterial activities (Schreiner *et al*. 2022; Yu *et al*. 2023). In this connection, we have proposed to use *Camellia oleifera* tea seed saponin for the fabrication of AgNPs by merging both biological and chemical approaches. In this study, new fabrication methods of AgNPs have been proposed through a combination of tea seed saponin extraction (*Camellia oleifera*) as a biological non-ionic surfactant with a cationic surfactant as co-surfactant. The strategies using *Camellia oleifera* tea seed saponin involve co-reducing and co-stabilising agents to reduce the use of synthetic chemical reagents and contribute to the sustainable development of the resources. As-prepared AgNPs exhibit antibacterial activity and initial potential for application in agriculture.

## MATERIALS AND METHODS

### Materials

Silver nitrate (AgNO_3_, 99%, Sigma-Aldrich, USA), cetyltrimethylammonium chloride (CTAC, 99%, Daejung), L-ascorbic acid (Xilong, China), ammonium hydroxide (NH_4_OH, 25%–28%, Daejung Chemical & metals, South Korea), tea seed saponin powder (30% saponin, China) and Mueller Hinton Agar (MHA, HiMedia Laboratory, India) were used without further purification.

### Fabrication of Silver Nanoparticles

*Camellia oleifera* tea seed saponin was first extracted by solid-liquid extraction according to a previous report, with modifications for optimisation (Yu & He 2018). Briefly, a defined amount of commercial tea seed powder (containing 15% saponins) was mixed with distilled water at a solid-to-liquid ratio of 1:6. The mixture was incubated at 80°C with continuous stirring for 6 h. The extract was subsequently separated by centrifugation at 5,000 rpm for 5 min and filtered through filter paper to obtain the final liquid extract.

Then, three different mixtures (total of 1.2 g) of saponin-to-CTAC ratios were prepared as series 1:0 (1.2 g/0 g), 7:3 (0.84 g/0.36 g), 1:1 (0.6 g/0.6 g), and 3:7 (0.36 g/0.84 g). Each mixture was dissolved in 30 mL of water by stirring for 5 min. The mixture of AgNO_3_ (0.074 M) and NH_4_OH (0.1 M) in water was slowly added to the co-surfactants solution and kept stirring for 10 min. Then 15 mL of L-ascorbic acid (0.4 M) in water was added. The reaction solution was heated to 70°C with a rate of 3°C/min–4°C/min under vigorous stirring for 1.5 h.

### Antibacterial Activity

Antibacterial activity was performed in *E. coli* (ATCC 25922) using two methodologies. Regarding the first method, AgNPs were mixed with a bacterial suspension and then spread to culture on agar plates to assess bacterial growth. The second method was the disk diffusion approach (Cunha *et al*. 2016). The AgNPs amount of 20 μL of series concentration of 0.08 ppm, 0.8 ppm, 8 ppm, 80 ppm and 800 ppm was used in both methods. The positive control was performed using ciprofloxacin (5 μg). In the disk diffusion approach, a sterile cotton swab was used to spread bacteria at a density equivalent to 10^7^ CFU/mL on a petri plate (Mueller-Hinton medium). Paper discs impregnated with 20 μL of AgNPs solutions were placed on the as-cultured plate, then incubated at 37°C for 24 h. The antibacterial activity of AgNPs was obtained by measuring the diameter of the inhibition zone around the paper disc. In addition, AgNPs solutions with different concentrations were also incubated with the bacteria (10^7^ CFU/mL) and then spread on the petri plate to obtain the growth behaviour.

### Characterisation

The morphology of AgNPs was obtained by scanning electron microscopy (SEM, JEOL JSM-7500F) and transmission electron microscopy (TEM, HITACHI H-710). Particle size and zeta (ξ) potential were also analysed by DLS (ZetaPALS, Brookhaven Instruments Co., USA) and Digimizer software. Optical properties were obtained by UV-vis spectroscopy. The bacterial density was supported by automated cell count (LUNA-II, Logos Biosystem, South Korea).

## RESULTS

**FIGURE 1:**
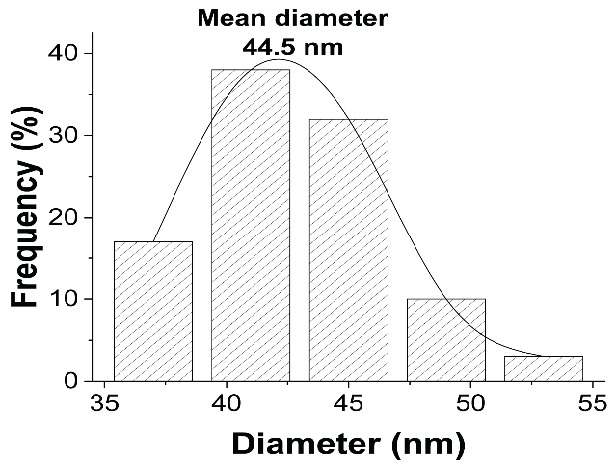
Particle size histogram of AgNPs.

**FIGURE 2:**
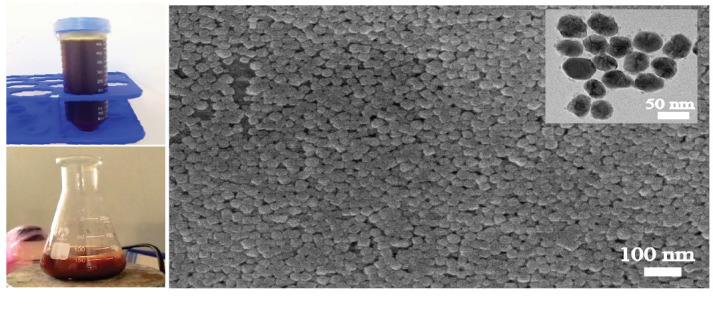
(a) Digital images and (b) SEM of AgNPs. The inset image is TEM of AgNPs.

**FIGURE 3:**
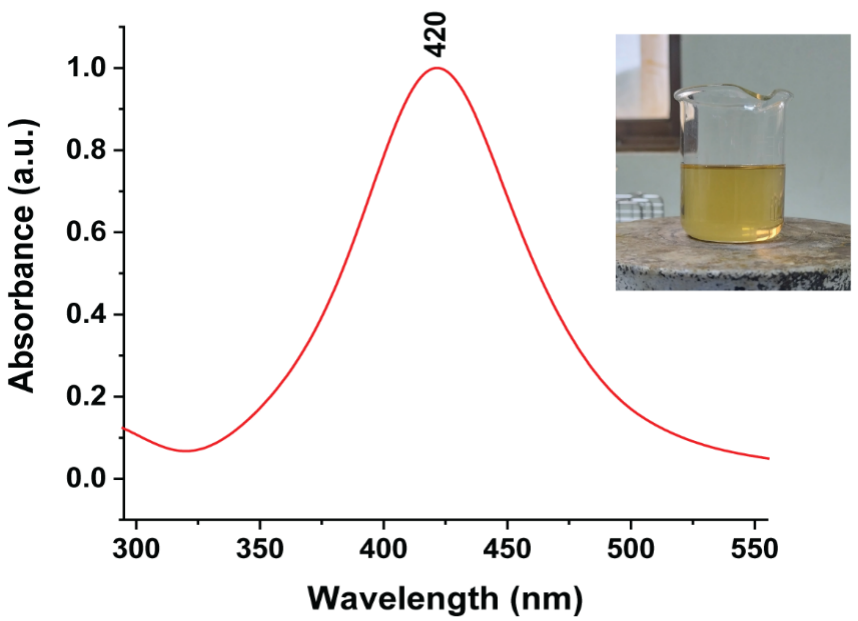
UV-vis absorbance spectrum of AgNPs suspension in water. The visual image shows the typical bright yellow colour of AgNPs solution.

The particle sizes of AgNPs synthesised using different saponin-to-CTAC ratios (1:0, 7:3, 1:1, and 3:7) were 82.2 ± 2.5 nm, 122.8 ± 5.2 nm, 44.5 ± 3.8 nm and 48.5 ± 1.5 nm, respectively. As-prepared AgNPs at ratios of 1:1 and 3:7 exhibited relatively small particle sizes, ranging from 40.5 nm to 48.5 nm, which were significantly smaller compared to those synthesised at ratios of 1:0 and 7:3. Particularly, saponin-to-CTAC ratios of 1:0 performed an almost neutral charge of –2.3 mV. The ξ-potential was altered to increase proportionally with CTAC concentrations. At a 1:0 ratio, the AgNPs displayed an almost neutral charge (–1.59 ± 2.98 mV), which progressively increased to 18.11 ± 1.63 mV (7:3), 30.63 ± 1.29 mV (1:1) and 44,66 ± 1.96 mV (3:7). It was observed that the ξ-potential measurements also revealed notable differences among the formulations, with the charge surface increasing correlation with the amount of CTAC, indicating the integration and combination of the two surfactants. Based on the results, AgNPs synthesised at the 1:1 saponin-to-CTAB ratio were selected for subsequent experiments due to their optimal particle size 44.5 ± 3.8 nm (Table 1) and high stability (ξ-potential of about 30 mV). Fig. 1 shows the histogram diagram of particle size distribution with a mean diameter of 44.5 ± 3.8 nm. Fig. 2 displays the digital, SEM and TEM images of as-prepared AgNPs. AgNPs were well suspended in water with a clear yellow as the typical colour of silver nanoparticles. The UV-vis spectra were exhibited at ~420 nm (Fig. 3). In this work, the formulation of AgNPs through a reduction reaction induced by glucose as a reducing agent, whereas synergist co-surfactant of saponin extraction solution and synthetic surfactant allows the stability of AgNPs. The combination of a bio-surfactant and an ionic surfactant enhances thermodynamic properties, surface tension and surface distribution depending on the properties of the ionic surfactant, such as alkyl chain length and surfactant ratios (Bagheri & Khalili 2017).

**FIGURE 4:**
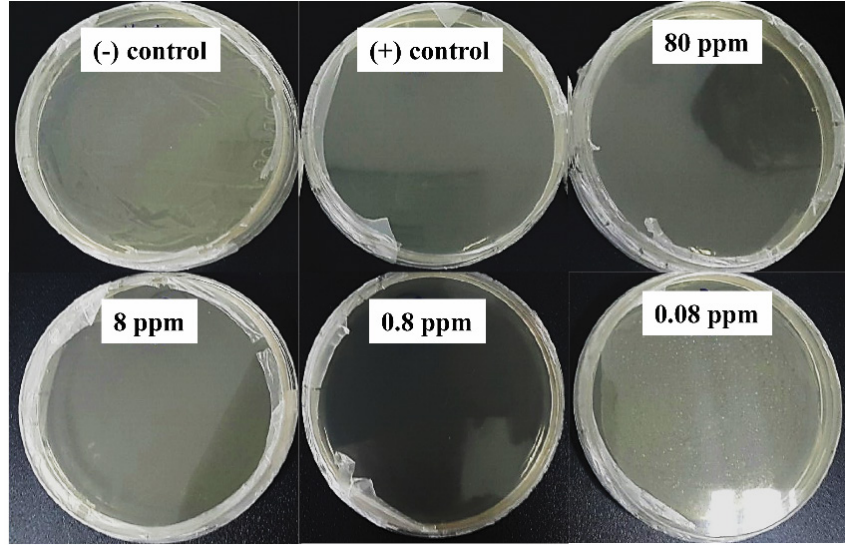

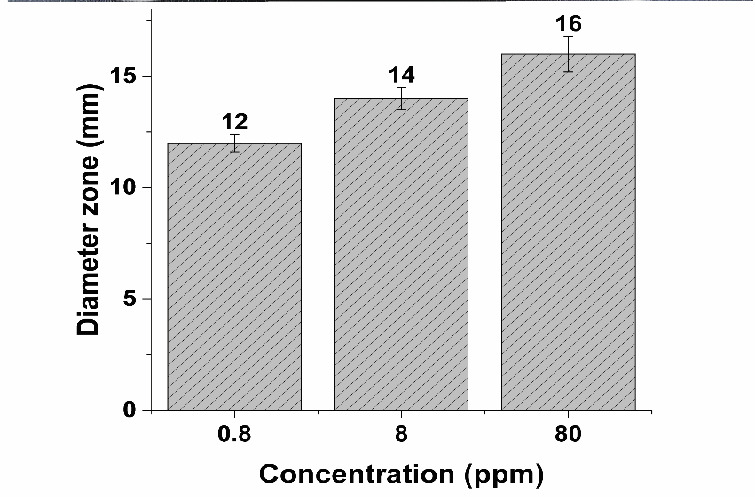

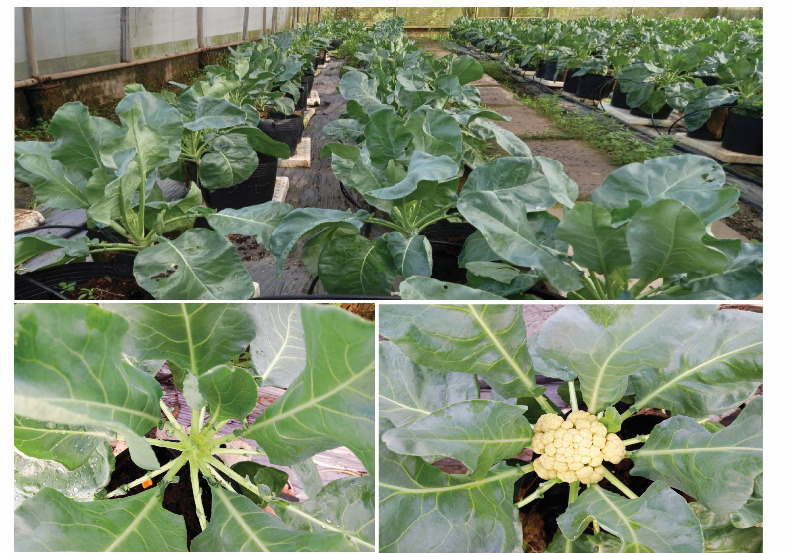
(a) Anti-bacterial behaviour of as-prepared AgNPs against E. coli ATCC 25922 as assessed by bacterial growth with minimum inhibitor at 0.08 ppm. (b) Diameter of the inhibition zone upon different concentrations of AgNPs, with minimum inhibitor at 0.8 ppm. (c) Young broccoli (*Brassica oleracea*) treatment with as-prepared AgNPs showing growth and development of plants with no leaf burn and no phytotoxicity.

As-prepared AgNPs were used to demonstrated antibacterial acitivity at concentrations of 80 ppm, 8 ppm, 0.8 ppm and 0.08 ppm were used to evaluate antibacterial activity. The AgNPs inhibited *E. coli* in assess bacteria growth method at a minimum effective concentration of 0.08 ppm (Fig. 4a), which was notably more potent compared to disk diffusion assays, where the minimum inhibitory concentration was observed at 0.8 ppm (Fig. 4b). As shown in Fig. 4b, *E. coli* has been successfully inhibited at the AgNPs concentration of 80 ppm, 8 ppm and 0.8 ppm. It was obtained that the increase in AgNPs concentrations leads to an increase in antibacterial activities. The diameter of the inhibition zone was 12 ± 0.5 mm, 14 ± 0.3 mm and 16 ± 0.6 mm according with the AgNPs concentration of 0.8 ppm, 8 ppm and 80 ppm. An initial trial of AgNPs suspension treatment in young broccoli (*Brassica oleracea*) was carried out to test whether as-prepared AgNPs damage plants as well as plant protection. AgNPs suspension of 0.8 ppm was watering both sides of the leaves for 14 days (frequency 2 times/week). The result shows that the as-prepared AgNPs did not damage plants. After 2 weeks, the plants grew and developed well, the leaves were smooth green and not burnt and no diseases appeared (Fig. 4c). Initial results indicate the potential application of as-prepared AgNPs in the formulation and development of products for plant protection. However, further studies should be carried out on various plant species as well as different period stages of plant growth. The assessment of environmental impact, clinical and sub-clinical studies of AgNPs also need to be carefully studied together with its strategies design and applications.

## DISCUSSION

The hydrophilic functional groups surrounding surfactant molecules help to stabilise the colloidal particle structure (Ebrahiminezhad *et al*. 2017). The role of surfactants of bioactive substances such as saponins and glycosides has proved not only positive in the formation of well-defined structures of particles (Mikhailova 2020) but also has a capacity of antibacterial, anti-inflammatory and antioxidant (Khodeer *et al*. 2023; Shoaib *et al*. 2024). Camellia oleifera mainly contains sapogenins, saccharides and organic acids. Saponin has the ability of antibacterial, anti-inflammatory and antioxidant (Dong *et al*. 2020; Khan *et al*. 2022; Singh *et al*. 2024). Evaluation of the surface activity and critical micelle concentration (CMC) forming ability of saponin showed that when esterified, tea saponin ester had significantly better surface activity. At the same time, the foaming ability, stability and emulsifying ability of saponin compared with other surfactants (such as non-ionic decyl glucoside, amphoteric cocoamido propyl hydroxy sulfobetaine and anionic ammonium laureth sulfate) showed that the foaming ability of tea residue saponin was weaker than that of ionic surfactants but significantly better than that of other surfactants. Therefore, saponin can be applied in food science to replace synthetic chemical surfactants, contributing to sustainable development resources (Singh *et al*. 2024; Zhang *et al*. 2023).

Currently, the exact antibacterial properties of AgNPs are still unclear. However, several of the hypotheses AgNPs mode of action have been proposed (Yin *et al*. 2020). AgNPs could release Ag^+^ that can adhere to penetrate the membrane and cytoplasm of bacterial cells through electrostatic attraction and affinity with other biomolecules, increasing permeability and causing the disruption of the bacterial envelope (More *et al*. 2023; Salleh *et al*. 2020). In addition, AgNPs and other related substances such as Ag^+^ and ROS from AgNPs have the ability to intercalate DNA to disrupt DNA replication or they can even directly destroy bacterial cells. In some cases, AgNPs may accumulate in the cell wall and cause membrane denaturation, leading to penetration and description of cell wall structure or cell lysis (Yin *et al*. 2020). AgNPs can also disrupt bacterial cell signaling pathways, which can lead to apoptosis and inhibit bacterial cell proliferation (Abdelgadir *et al*. 2024). Therefore, the implementation of fabrication and application of AgNPs for the prevention and treatment of pathogens has been a concern in many fields, including agriculture for crop protection. The antibacterial behaviour of AgNPs depends on the size, shape, surface and physical and chemical properties that allow AgNPs can interact with microbial cells (Menichetti *et al*. 2023). AgNPs have been proven to have a high potential for application in agriculture against insects and pathogens in crops.

## CONCLUSION

In this work, the combination of natural surfactant and chemical co-surfactant has been successful in the fabrication of AgNPs that can be beneficial in the design and implementation of antibacterial applications through various modes of action. The strategies of using tea seed saponin are as co-reducing and co-stabilising agents to certainly reduce the use of chemical substances and open the way to contribute to sustainable development.

## Figures and Tables

**TABLE 1:** Particle size distribution of AgNPs2.

No.	Diameter (nm)	Frequency (%)
1	37–40	17
2	41–44	38
3	45–48	32
4	49–52	10
5	53–54	3

*Note:* Min: 37.5 nm; Max: 54.5 nm.
